# Usefulness of dual-axis rotational coronary angiography in primary percutaneous coronary intervention for patients with ST-elevation myocardial infarction

**DOI:** 10.1007/s00380-020-01738-2

**Published:** 2020-12-04

**Authors:** Hideaki Suwa, Yota Koyabu, Taichi Adachi, Akira Kawai, Kazuhiko Kotani, Shigeru Toyoda, Teruo Inoue, Toru Kato

**Affiliations:** 1 Department of Cardiovascular Medicine, National Hospital Organization Tochigi Medical Center, 1-10-37, Nakatomatsuri, Utsunomiya, Tochigi 320-8580 Japan; 2 Department of Cardiovascular Medicine, Dokkyo Medical University, Mibu, Tochigi Japan; 3 Department of Radiology, National Hospital Organization Tochigi Medical Center, Utsunomiya, Tochigi Japan; 4 Division of Community and Family Medicine, Center for Community Medicine, Jichi Medical University, Shimotsuke, Tochigi Japan; 5 Department of Clinical Research, National Hospital Organization Tochigi Medical Center, Utsunomiya, Tochigi Japan

**Keywords:** ST-elevation myocardial infarction, Primary percutaneous coronary intervention, Contrast medium, Radiation exposure, Rotational coronary angiography

## Abstract

Several studies have shown that dual-axis rotational coronary angiography (DARCA) reduces contrast medium volume and radiation exposure compared to conventional coronary angiography (CCA). However, there are no studies comparing the safety and usefulness of DARCA in primary percutaneous coronary intervention (PCI) for patients with ST-elevation myocardial infarction (STEMI). The aim of this study was to investigate the effects of DARCA on contrast medium volume, radiation exposure, time course of treatment, and adverse events in primary PCI for patients with STEMI. A total of 82 patients undergoing primary PCI were included in this study. Subjects were propensity matched to 41 patients in the CCA group and 41 in the DARCA group. Data were retrospectively collected from in-patient medical records and the contrast medium volume and radiation exposure (dose-area product, DAP) during the PCI procedure was compared between the two groups. Contrast medium volume [100.0 (82.5–115.0) vs 110 (102.5–127.5) ml,* p* = 0.018, *r* = 0.26] and DAP [113.4 (74.3–141.1) vs 138.1 (100.5–194.7) Gy cm^2^, *p* = 0.014, *r* = 0.27] were significantly lower in the DARCA group, compared with the CCA group. Door to device time (68.7 ± 26.1 vs 76.5 ± 44.2 min, *p* = 0.33) were comparable between the two groups. There were no adverse events requiring treatment reported in either groups. DARCA may reduce contrast medium volume and radiation exposure in primary PCI for patients with STEMI, and can be used safely, without delaying reperfusion of the infarct-related coronary artery.

## Introduction

Coronary angiography plays an important role in the diagnosis and treatment of coronary artery disease since first performed by Sones in 1959 [1]. As catheter and angiographic systems have evolved over time, their safety and diagnostic accuracy have also improved. However, conventional coronary angiography (CCA) requires multidirectional views of both the left and right coronary arteries. It is also well known that the use of contrast medium and radiation can lead to adverse events with increased use [2–6]. Therefore, various techniques have been devised to reduce contrast medium volume and radiation exposure while maintaining diagnostic accuracy. Coronary artery rotation imaging, one of such techniques, has been utilized since 1998 [7, 8]. Coronary artery rotation imaging has since evolved to the dual-axis rotational coronary angiography (DARCA) system. Numerous studies have demonstrated that DARCA reduces contrast medium volume and radiation exposure while ensuring diagnostic accuracy compared to CCA [9–14]. Most studies regarding DARCA, however, have focused on its use in scheduled coronary angiography and elective percutaneous coronary intervention (PCI) for patients with chronic coronary artery disease or non-ST-elevation myocardial infarction (NSTEMI). There are currently no studies comparing the safety and usefulness between DARCA and CCA in primary PCI for patients with ST-elevation myocardial infarction (STEMI). We hypothesized that the use of DARCA reduces contrast medium volume and radiation exposure in primary PCI for patients with STEMI while ensuring diagnostic accuracy, without affecting door to device time.

## Materials and methods

### Study design and subjects

**Fig. 1:**
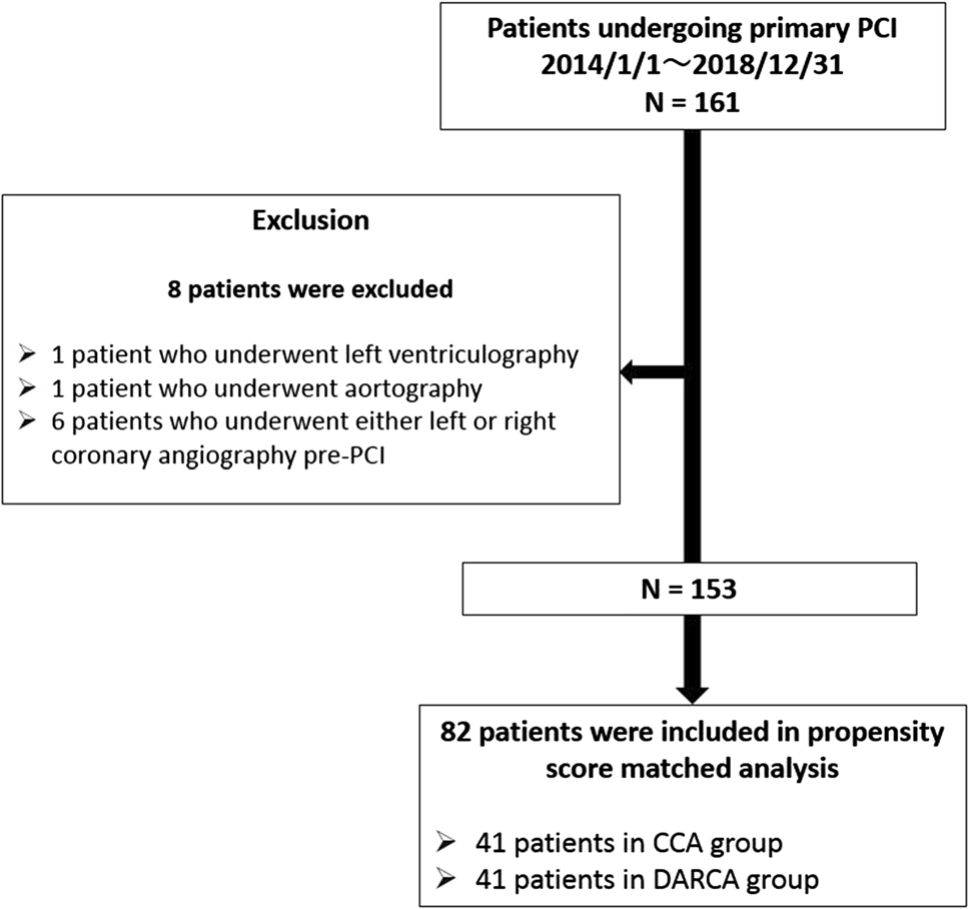
Flow chart of the present study. *PCI* percutaneous coronary intervention, *STEMI* ST-elevation myocardial infarction, *NSTEMI* non-ST-elevation myocardial infarction, *CCA* conventional coronary angiography, *DARCA* dual-axis rotational coronary angiography

This was a single-center, retrospective, cross-sectional observational cohort study. The charts of 161 patients with STEMI who underwent primary PCI at the National Hospital Organization Tochigi Medical Center during the period from January 1, 2014 to December 31, 2018 were reviewed. The PCI procedure included pre-PCI coronary angiography for both left and right coronary arteries. Two patients who underwent aortography and/or left ventriculography during the procedure in addition to coronary angiography and 6 patients who underwent either left or right coronary angiography prior to PCI were excluded. The remaining 153 patients were included in our analysis (Fig. 1). The study followed the Ethical Guidelines for Medical and Health Research Involving Human Subjects [15], and the study protocol was approved by the Ethics Review Committee of National Hospital Organization Tochigi Medical Center. The authors have conformed to institutional guidelines and those of the American Physiological Society.

### Diagnosis of STEMI and primary PCI procedure

Diagnosis of STEMI was determined when there was evidence of elevated cardiac troponin values (troponin T) above the 99th percentile upper reference limit and with at least one of the following clinical evidence: symptoms of myocardial ischemia; new ischemic ECG changes; and development of pathological Q waves; imaging evidence of new loss of viable myocardium or new regional wall motion abnormality in a pattern consistent with an ischemic etiology [16]. Emergent cardiac catheterization procedure was performed per the 2017 European Society of Cardiology guidelines for the management of acute myocardial infarction [17]. Informed consent for primary PCI was obtained from each patient while in the emergency department, after the diagnosis of STEMI. All patients received dual-antiplatelet therapy with 200 mg of Aspirin and with 20 mg of Prasugrel or 300 mg of Clopidogrel as a loading dose stipulated by insurance adaptation in Japan [18].

Cardiac catheterization was performed using a single-plane cine-angiography apparatus, Allura Xper FD 10/20 digital X-ray system (Philips Healthcare, Best, the Netherlands), and was performed by the transradial or transfemoral approach. Pre-PCI coronary angiography was performed using 5-Fr diagnostic catheters. CCA or DARCA was performed by the operator’s discretion regardless of the culprit vessel and non-culprit vessel. All patients received heparin bolus and patients with stable hemodynamics received isosorbide-dinitrate injections into the right and the left coronary arteries prior to starting angiography. We used iopamidol as a contrast medium and its injection was done manually, rather than using an automatic injector. PCI was performed using 6- or 7-Fr guiding catheters. In all patients, drug-eluting stents were placed to the culprit lesion under intravascular ultrasound image-guiding. Thrombus aspiration and distal protection were added as appropriate. There were patients with unstable hemodynamics and cardiogenic shock from the beginning at emergency room. Temporary pacing and/or intra-aortic balloon pumping were used if necessary, per the discretion of the cardiologist.

### Cohort clinical characteristics

Obesity was defined as body mass index ≥ 25 kg/m^2^. Hypertension was defined as systolic blood pressure ≥ 140 mmHg or diastolic blood pressure ≥ 90 mmHg. Diabetes was defined according to the Japanese Diabetes Society: fasting blood glucose ≥ 126 mg/dL or random blood glucose ≥ 200 mg/dL, or need for anti-diabetic drugs. Dyslipidemia was defined per the Japanese atherosclerosis society: low-density lipoprotein-cholesterol ≥ 140 mg/dL, triglyceride ≥ 150 mg/dL or high-density lipoprotein-cholesterol < 40 mg/dL or need for anti-dyslipidemic drugs.

### XperSwing characteristics

**Fig. 2:**
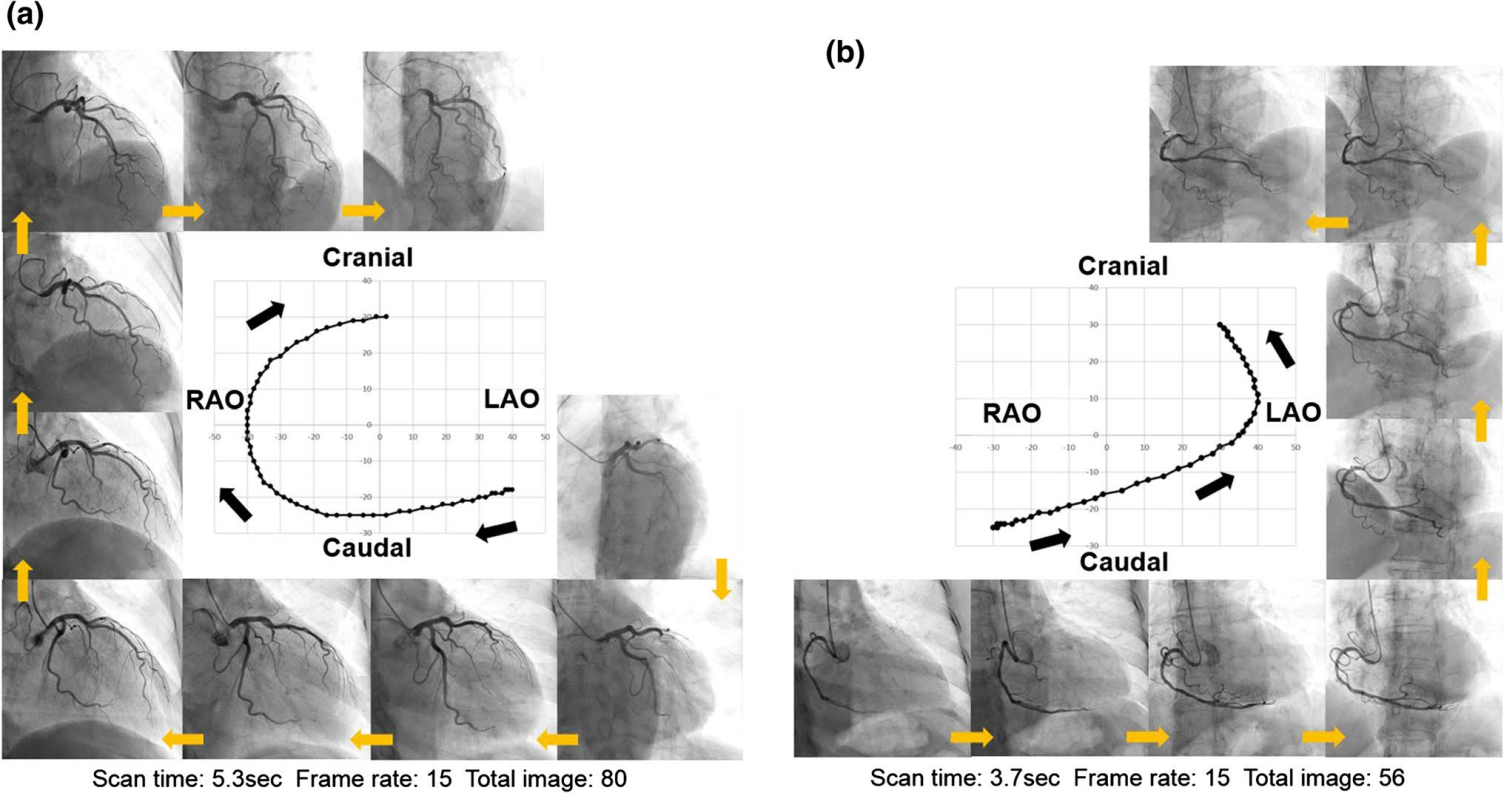
**a** DARCA of the left coronary artery. Multiple sequential (LAO to RAO and caudal to cranial) representative frames of the DARCA acquisition are displayed. **b** DARCA of the right coronary artery. Multiple sequential (RAO to LAO and caudal to cranial) representative frames of the DARCA acquisition are displayed. *DARCA* dual-axis rotational coronary angiography, *LAO* left anterior oblique, *RAO* right anterior oblique

XperSwing is a system of DARCA by Philips, in which the C-arm radiates X-rays while moving around two axes of pre-programmed Cranio-Caudal direction and left anterior oblique (LAO)-right anterior oblique (RAO) direction. One of the advantages of DARCA is it can take images from a plurality of directions that cannot be imaged by CCA with one injection of contrast medium. Instead, DARCA is required that one imaging time is longer, so that the amount and time of one injection of the contrast medium is longer than that of CCA. DARCA need to check the two views (frontal and LAO) and perform a test run to adjust the patient’s position prior to coronary angiography. The C-arm moves so as not to contact the fixed patient, the pre-programmed imaging angle is set shallow. Thus, in some cases, it may be necessary to add deep-angle views. For the left coronary artery, a total of 80 images are continuously captured at Frame rate 15 (frames per second) while the C-arm moves from LAO-Caudal through Caudal, RAO, Cranial direction and finally to LAO-Cranial in 5.3 s (Fig. 2a). Imaging of the right coronary artery is programed to continuously capture a total of 56 images at Frame rate 15 (frames per second) while the C-arm moves from RAO-Caudal through Caudal, LAO direction and finally to LAO-Cranial in 3.7 s (Fig. 2b).

**Fig. 3:**
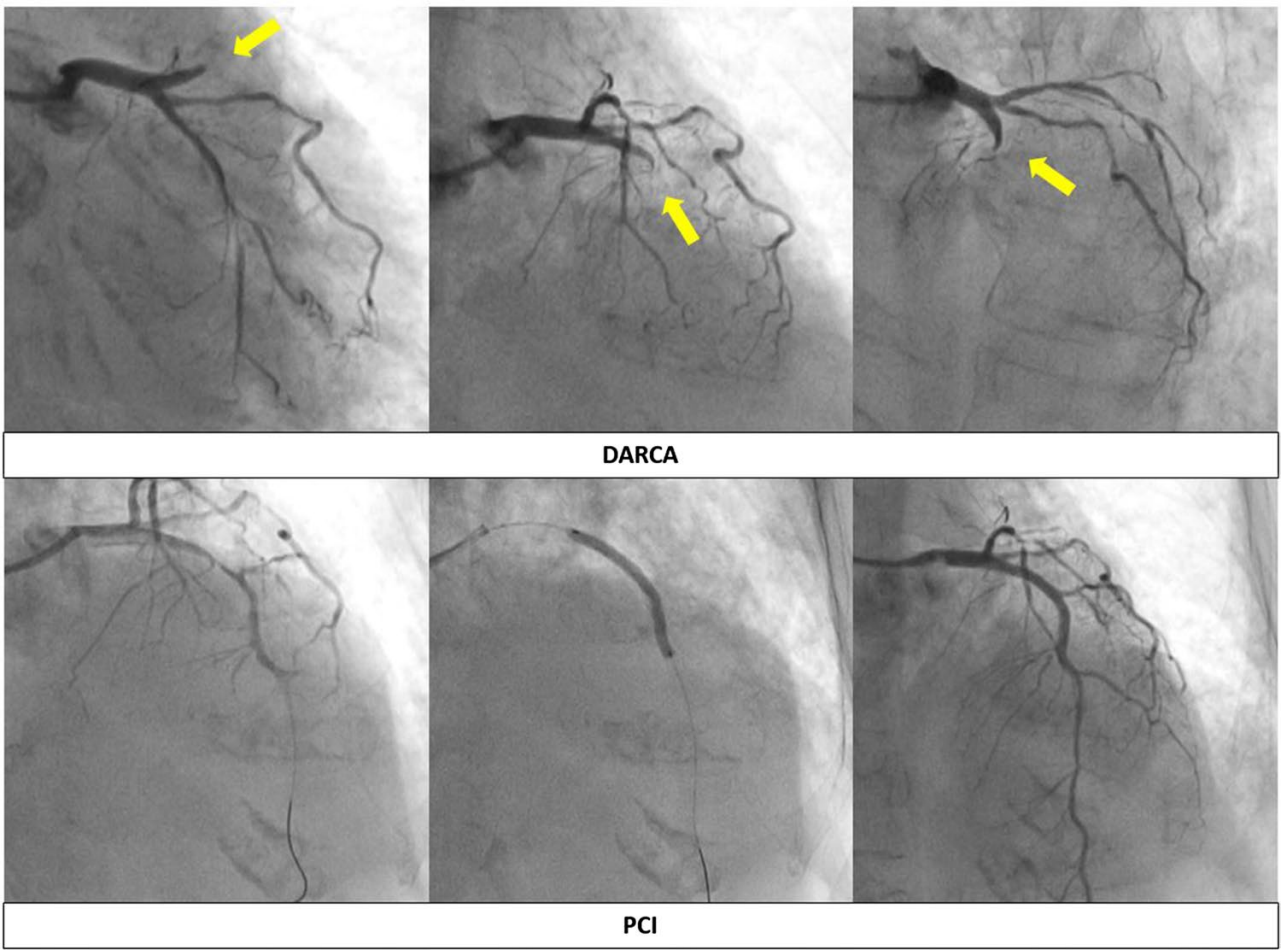
A case of STEMI diagnosed by DARCA. DARCA demonstrated multidirectional views of LAD proximal occlusion by only single cine-angiography. *DARCA* dual-axis rotational coronary angiography, *STEMI* ST-elevation myocardial infarction, *PCI* percutaneous coronary intervention, *LAD* left anterior descending artery

Figure 3 shows a case of occlusion lesions of left anterior descending artery (LAD) diagnosed using DARCA (Fig. 3).

### Study groups

The cohort was divided into two groups: DARCA group and CCA group. In the DARCA group, DARCA was used at least once in either the left or right coronary angiography using XperSwing, and additional coronary angiography was performed without using XperSwing if necessary. Thus, the DARCA group included both patients in whom DARCA was used combined with CCA and those undergoing DARCA alone. In the CCA group, CAG was performed without using XperSwing. Thus, the CCA group consisted of only patients undergoing CCA alone. In this study, DARCA was used during pre-PCI coronary angiography, but not after the following PCI procedure was started. Usage of the DARCA was based on the operator’s decision.

### Outcomes of this study

The primary outcome of this study was contrast medium volume and radiation exposure during overall PCI-related procedures, including pre-PCI coronary angiography and the following PCI procedure. Contrast medium volume was measured as a total amount from sheath insertion to the end of all procedures. Radiation exposure was assessed as the dose-area product (DAP) measured with the X-ray system from the start to end of procedure. The DAP is the product of the dose value of the incident radiation and the irradiated field size and is expressed in Gy·cm^2^. The DAP meters (Diamentor, PTW-Freiburg, Germany/KermaX-plus, Wellhöfer, Germany) were integrated into the X-ray systems. DAP values, as well as the fluoroscopy time, were entered into a dedicated electronic database linked to the catheterization registry database. Secondary outcomes are as listed: ① Angiographic runs pre-PCI: number of angiographic imagings before coronary artery guidewire insertion; ② Door to device time: time interval from hospital visit to guidewire passage through the culprit lesion; ③ Coronary angiography to device time (CAG to device time): time interval from initiation of coronary angiography to guidewire passage through the culprit lesion; ④ Adverse events associated with coronary angiography: bradycardia and other symptomatic arrhythmias (supraventricular and/or ventricular tachycardia requiring treatment, ventricular fibrillation), shock and cardiopulmonary arrest associated with coronary angiography prior to PCI; and ⑤ contrast-induced nephropathy: defined as ≥ 25% or ≥ 0.5 mg/dl increase in serum creatinine level at 48 h post-PCI [19].

### Statistical analysis

For comparisons between the two groups of DARCA and CCA, we performed propensity score matching analysis using “Greedy-digit matching” without replacement to control for patients’ background clinical characteristics. Matching was performed using a 1:1 matching protocol with a caliper width equal to 0.2 of the standard deviation of the logit of the propensity score. The standardized difference of < 10.0% for a given covariate was considered as a relatively small imbalance. After verifying the sample distribution, intergroup comparisons of continuous variables were performed using unpaired Student *t* test for normally distributed data or Mann–Whitney test for skew-distributed data. Categorical variables were compared by Chi-square test. Data were expressed as mean ± standard deviation for continuous variables and number and percentage for categorical variables. Statistical significance was set at *P* values < 0.05. All the analyses were performed with SPSS software, version 25.0 (IBM Corporation, Somers, New York, USA).

## Results

### Propensity score matching

Propensity score matching was performed for the 153 patients (63 in CCA group, 90 in DARCA group) in our study cohort. After adjusting for age, gender, obesity, underlying hypertension, diabetes mellitus, hyperlipidemia, indication for PCI, Killip classification, culprit lesion, lab data, catheter approach, and guiding catheter diameter, we were left with 41 patients in each group (CCA and DARCA) (Fig. 1). After propensity score matching, baseline clinical characteristics were similar between the CCA group and the DARCA group (Table 1). Among the 41 DARCA patients, 30 patients (73%) underwent DARCA combined with CCA and the remaining 11 (27%) underwent DARCA alone.

**Table 1:** Baseline characteristics before and after propensity score matching

	Before propensity score matching				After propensity score matching			
	(*n* = 153)				(*n* = 82)			
	CCA	DARCA	*P* value	Standardized difference	CCA	DARCA	*P* value	Standardized difference
	(*n* = 63)	(*n* = 90)			(*n* = 41)	(*n* = 41)		
Age, years	69.9 ± 13.3	68.7 ± 12.5	0.58	0.09	69.1 ± 14.9	70.6 ± 12.6	0.62	0.11
Male gender, n (%)	46 (73.0)	68 (75.6)	0.72	0.06	32 (78.1)	28 (68.3)	0.32	0.22
Obesity, n (%)	26 (41.9)	27 (30.0)	0.13	0.25	16 (39.0)	12 (29.3)	0.35	0.21
Hypertension, n (%)	42 (66.7)	68 (75.6)	0.23	0.20	25 (61.0)	29 (70.7)	0.35	0.21
Diabetes mellitus, n (%)	30 (47.6)	37 (41.1)	0.43	0.13	18 (43.9)	18 (43.9)	1	0.00
Dyslipidemia, n (%)	34 (54.0)	54 (57.5)	0.46	0.07	24 (58.5)	24 (58.5)	1	0.00
Killip, n (%)			< 0.01				0.55	
1, 2	53 (84.1)	87 (96.7)		0.44	39 (95.1)	40 (97.6)		0.13
3, 4	10 (15.9)	3 (3.3)		0.44	2 (4.9)	1 (2.4)		0.13
Culprit lesion, n (%)			0.09				0.51	
Left main trunk	3 (4.8)	0 (0)		0.32	0 (0)	0 (0)		–
Left anterior descending artery	23 (37.1)	42 (46.7)		0.20	16 (39.0)	21 (51.2)		0.25
Left circumflex artery	13 (21.0)	15 (16.7)		0.11	10 (24.4)	7 (17.1)		0.18
Right coronary artery	23 (37.1)	33 (36.7)		0.01	15 (36.6)	13 (31.7)		0.10
Lab data								
Creatinine, mg/dL	1.09 ± 0.84	0.94 ± 0.41	0.13	0.24	0.94 ± 0.37	0.96 ± 0.47	0.79	0.05
Troponin T, ng/mL	1.12 ± 2.56	0.83 ± 1.52	0.40	0.14	0.950 ± 1.766	0.789 ± 1.498	0.66	0.10
Catheter approach site, n (%)			< 0.0001				1	
Radial approach	33 (52.4)	79 (87.8)		1.48	32 (78.1)	32 (78.1)		0.00
Femoral approach	30 (47.6)	11 (12.2)		1.35	9 (21.9)	9 (21.9)		0.00
Guiding catheter diameter, n (%)			< 0.0001				0.29	
6 Fr	45 (71.4)	86 (95.6)		2.23	40 (97.6)	38 (92.7)		0.23
7 Fr	18 (28.6)	4 (4.4)		0.90	1 (2.4)	3 (7.3)		0.23

Values are mean ± standard deviations or number (percentage)

*CCA* conventional coronary angiography, *DARCA* dual-axis rotational coronary angiography

### Primary outcomes

Contrast medium volume during the primary PCI procedure was significantly lower in the DARCA group than in the CCA group (median [interquartile range] 100.0 [82.5–115.0] vs 110 [102.5–127.5] ml, *p *= 0.018, *r* = 0.26). DAP during the PCI procedure was also significantly lower in the DARCA group than in the CCA group (median [interquartile range] 113.4 [74.3–141.1] vs 138.1 [100.5–194.7] Gy cm^2^, *p* = 0.014, *r* = 0.27) (Table 2).

**Table 2:** Primary and secondary outcomes

	CCA	DARCA	*P* value
	(*n* = 41)	(*n* = 41)	
Primary outcome			
Contrast medium volume, ml	110.0 (102.5–127.5)	100.0 (82.5–115.0)	0.018
DAP, Gy cm^2^	138.1 (100.5–194.7)	113.4 (74.3–141.1)	0.014
Secondary outcomes			
Number of angiographic runs pre-PCI, n	6.7 ± 2.4	2.9 ± 1.1	< 0.0001
Door to device time, min	76.5 ± 44.2	68.7 ± 26.1	0.33
CAG to device time, min	14.8 ± 7.1	15.9 ± 9.5	0.56
Adverse event during procedure, n (%)			
Bradycardia	4 (10.0)	13 (31.7)	0.014
Symptomatic arrhythmia	0 (0)	0 (0)	–
Shock	0 (0)	0 (0)	–
Cardiac arrest	0 (0)	0 (0)	–
Contrast-induced nephropathy, n (%)	2 (4.9)	6 (14.6)	0.13

Values are mean ± standard deviations, medians (interquartile ranges) or number (percentage)

*CCA* conventional coronary angiography, *DARCA* dual‐axis rotational coronary angiography, *DAP* dose area product, *PCI* percutaneous coronary intervention, *CAG* coronary angiography

### Secondary outcomes

The number of angiographic runs for pre-PCI coronary angiography was significantly less in the DARCA group than in the CCA group (2.9 ± 1.1 vs 6.7 ± 2.4, *p* < 0.0001) (Table 2). Door to device time (76.5 ± 44.2 vs 68.7 ± 26.1 min, *p* = 0.33 and CAG to device time (14.8 ± 7.1 vs 15.9 ± 9.5 min, *p* = 0.56) were comparable between the two groups (Table 2). As for the adverse events associated with coronary angiography, only transient asymptomatic bradycardia was observed in both groups, and the incidence was more frequent in the DARCA group than in the CCA group (4 vs 13, *p* = 0.014) (Table 2). There was no significant difference in the incidence of contrast-induced nephropathy between the two groups (Table 2).

## Discussion

In this study, we examined the safety and usefulness of DARCA in primary PCI for patients with STEMI, with focus on contrast medium volume, radiation exposure, procedure time, and adverse events during the procedure compared to CCA. While there are data pertaining to DARCA in elective CAG and PCI, the literature is little regarding DARCA and acute myocardial infarction (AMI) [20, 21]. This is the first study to evaluate DARCA in primary PCI for patients with STEMI. Compared to the CCA group, the DARCA group required less contrast medium volume and received less radiation exposure. Door to device time and CAG to device time were comparable between the DARCA and CCA groups. Adverse events were also similar between the two groups except for transient asymptomatic bradycardia.

Although safety and usefulness of DARCA have been shown in scheduled PCI for chronic coronary artery disease and NSTEMI patients [11, 13, 14, 20–23], our study shows safety and usefulness in primary PCI, as well. We believe the significant reduction in contrast medium volume and radiation exposure in the DARCA is probably due to the fact that the number of angiographic runs for pre-PCI coronary angiography was small in DARCA cases. We should not neglect the accuracy of diagnostic coronary angiography by reducing the number of angiographic runs. DARCA can identify culprit lesions and evaluate lesion morphology from various angles in a single cine-angiography, which is considered to be very useful in developing treatment strategies for STEMI patients with unknown coronary arterial trees. In addition DARCA is easy to identify the imaging angle that accurately captures the culprit lesion. It is important to accurately captures the culprit lesion in PCI procedure. There were no diagnostic errors with DARCA and all patients performed DARCA were able to identify culprit lesions in first cine-angiography. On the other hand, left main trunk occlusion lesions and right coronary artery proximal occlusion lesions that do not require evaluation at multiple angles by DARCA may not only less effectiveness but also increase the contrast media and radiation exposure than CCA. If left main trunk occlusion lesions and right coronary artery proximal occlusion lesions can be confirmed in the first test shot, it is desirable to perform CCA. To target both avoiding increase in the number of angiographic runs and acquisition of high-quality imaging, however, DARCA combined with CCA would be promising [14]. Actually, in the present study, 73% of the patients in DARCA group underwent DARCA combined with CCA. In addition, it is controversial whether DARCA may lead to a shorten or delay in PCI procedure [8, 10, 22–26], our study found that there was no difference not only in the door to device time, but also in the CAG to device time, reflecting the procedure time, between the two groups of DARCA and CCA.

It is worth noting that procedure time in DARCA is also dependent on the skill level of the operator, and the learning curve for operating DARCA successfully has been well described [10, 20, 25, 27, 28]. However, it is not clear how long it takes to get DARCA running smoothly and to use DARCA safely in primary PCI. Based on our experience, if the operators become accustomed to using DARCA for diagnostic coronary angiography to some extent, application of DARCA for primary PCI procedure would be acceptable.

In patients with STEMI, particularly those with, or prone to, hemodynamic instability, there is concern about the safety for DARCA due to increased contrast medium utilization in one injection [10, 22, 29]. Regarding the adverse events in our study, however, no patients experienced hemodynamic collapse during the coronary angiography prior to PCI in any of our subjects. DARCA was also performed in safety at patients with Killip 4. The occurrence of bradycardia in CAG was more common in DARCA than CCA. We analyzed patients with bradycardia, there were no significant differences in the culprit lesions and the other parameters. Characteristic of patients with bradycardia were not clear. All bradycardia were temporary and asymptomatic, there was no problem in safety.

Contrast-induced nephropathy, likely to occur with increase in contrast medium volume, adversely lengthens hospital stay and increases hospital mortality in patients with acute coronary syndrome [2, 30]. Some studies have shown that DARCA reduced contrast-induced nephropathy in patients with AMI excluding STEMI [13, 21]. In this study, the incidence of contrast-induced nephropathy in the DARCA group did not differ from that in the CCA group. Thus, we believe that our study illustrates the safety of DARCA also from a perspective of risk for contrast-induced nephropathy.

It is necessary to examine the safety and efficacy of DARCA in a larger population using prospective studies for patients with STEMI undergoing primary PCI in the near future.

### Study limitations

We acknowledge the limitations of this study. First, in our study, the contrast medium volume was measured as a total amount from sheath insertion to the end of procedure and radiation exposure was assessed as total amount from the start to end of procedure. Essentially, these variables were assessed throughout all PCI-related procedures, limiting our ability to distinguish between the pre-PCI and PCI procedure settings. Second, in the case of DARCA, recommended contrast medium volume is 14–18 mL for left coronary artery and 7–10 mL for right coronary artery using an automatic injector. In this study, however, the operators manually injected the contrast medium because no automatic injector has been used in our hospital. As a result, up to 10 mL of contrast medium was infused into the left coronary artery, which was below the recommended amount for DARCA. Such a matter as manual contrast medium injection might affect the result of this study. Third, we used a single-plane angiographic system in this study, so we could not examine the difference between DARCA and CCA when the biplane system was used. The biggest limitation might be that two groups of DARCA and CCA were not randomized, since usage of the DARCA system was based on the operators’ decision. To resolve this issue, we used propensity-matching score to correct confounding factors.

## Conclusion

The present study shows that the use of DARCA in primary PCI for patients with STEMI allows for reduced total contrast medium volume and total radiation exposure compared to CCA alone, suggesting that DARCA may be used safely and without delaying the door to device time.
